# In sickness and in health: profiling the bovine fecal eukaryome using optimized 18S metabarcoding with a mammal-blocking primer

**DOI:** 10.3389/fvets.2026.1948426

**Published:** 2026-10-08

**Authors:** Iqra Zafar, Riku Sakurai, Yasuhiro Fukuda, Megumi Itoh, Atsushi Fujikura, Akira Goto, Kazuhiro Bandai, Kentaro Kato

**Affiliations:** 1 Laboratory of Sustainable Animal Environment, Graduate School of Agricultural Science, Tohoku University, Osaki, Miyagi, Japan; 2 Department of Veterinary Medicine, Obihiro University of Agriculture and Veterinary Medicine, Obihiro, Hokkaido, Japan; 3 Vets Support WA, Itoshima, Fukuoka, Japan; 4 Department of Veterinary Medicine, Faculty of Veterinary Medicine, Okayama University of Science, Imabari, Ehime, Japan; 5 Graduate School of Veterinary Science, Osaka Metropolitan University, Izumisano, Osaka, Japan

**Keywords:** calf diarrhea, *Cryptosporidium parvum*, dysbiosis, enteric protozoa, fecal mycobiota, host DNA contamination, host–parasite interactions, pathogen surveillance

## Abstract

**Introduction:**

Calf diarrhea, caused by the apicomplexan pathogen *Cryptosporidium parvum* (*C. parvum*), poses significant economic and production challenges to the livestock industry. While 16S rRNA sequencing has advanced understanding of the bovine bacterial gut microbiota, the eukaryotic community remains underexplored due to methodological constraints, notably host DNA contamination. This study aimed to optimize an 18S rRNA metabarcoding workflow using mammal-blocking primers for bovine fecal eukaryome profiling and to characterize eukaryotic community differences between healthy and naturally *C. parvum*-infected diarrheic calves.

**Methods:**

We compared four 18S rRNA primer sets (V8f/1510R, TAReuk454FWD1/TAReukREV3, 528F/706R, and 616F/1132R) with two mammal-blocking primers using fecal samples from healthy (*n* = 28) and *C. parvum*-positive diarrheic calves (*n* = 24) across Japan. The optimal primer-blocker combination was selected based on host-read depletion and taxonomic recovery. Using this optimized workflow, we characterized and compared the fecal eukaryotic microbiota between healthy and *C. parvum*-infected calves via 18S rRNA amplicon sequencing.

**Results:**

The V8 primer-blocker demonstrated the most consistent host suppression (30–50% reduction), unmasking diverse eukaryotic taxa while improving evenness and preserving community richness. *Cryptosporidium* dominated the diarrheic group and was confirmed as *C. parvum* via nested GP60 PCR. Infected calves were associated with lower eukaryotic richness, with reduced Chao1 richness (*p* < 0.00001) and Shannon diversity (*p* < 0.041), and distinct community composition (Bray-Curtis: *R*^2^ = 0.145, *p* = 0.001; Jaccard: *R*^2^ = 0.060, *p* = 0.001). After accounting for geographic variation, clinical status remained significantly associated with community composition (PERMANOVA, *R*^2^ = 0.129, *p* = 0.0001), whereas geography was not significant (*p* = 0.180). LEfSe identified *Cryptosporidium* (LDA score = 5.6) as the strongest discriminator in the Crypto group, while *Kazachstania* (4.35), *Giardia* (4.0), and *Aspergillus* (0.8) were enriched in healthy calves, reflecting complex interkingdom interactions.

**Conclusion:**

The optimized V8-mammal-blocking strategy provides an effective framework for bovine fecal eukaryome profiling by reducing host interference and improving recovery of clinically relevant microeukaryotes. *C. parvum* infection is associated with significant restructuring of the fecal eukaryotic community. This workflow strengthens livestock disease surveillance, facilitates future epidemiological studies, and advances our understanding of complex host–parasite and microeukaryome interactions.

## Introduction

Neonatal calf diarrhea remains a significant economic burden to the global cattle industry, characterized by high morbidity and potential mortality in pre-weaned animals (1, 2). Among the various causes, cryptosporidiosis is particularly prevalent in young calves. This disease causes severe enteritis, impaired growth, and increased mortality, which poses significant challenges to livestock production systems (3). Beyond its veterinary importance, the apicomplexan parasite *Cryptosporidium parvum* (*C. parvum*) also has significant zoonotic potential (4), highlighting the need for improved diagnostic and surveillance approaches.

The gut microbiota of cattle plays a critical role in host health, nutrient metabolism, and immune function (5). Disruptions to this microbial community during enteric disease are frequently associated with acute dysbiosis and the proliferation of secondary pathogens (6–8). Although often overlooked, parasitic infections can substantially contribute to dysbiosis by altering host immune responses and reshaping the composition of the microbial community (9). High-throughput sequencing has greatly advanced our understanding of gut microbial communities. However, most studies have focused primarily on the bacterial populations using 16S rRNA gene sequencing (10, 11). Consequently, the broader eukaryotic microbiota, including commensal protozoa, fungi, and helminths, remains underexplored due to methodological limitations (12). There is growing recognition that microeukaryotes contribute to intestinal homeostasis and disease processes through complex interactions with both the host and other microorganisms (13–15). Apicomplexan parasites exist within this broader eukaryotic ecosystem. Importantly, interactions between these parasites and co-occurring microbial eukaryotes may influence parasite colonization, persistence, and pathogenicity (16). Therefore, characterization of the intestinal eukaryome may provide valuable insights into parasite ecology, host–parasite interactions, and disease progression in livestock.

Characterization of complex eukaryotic communities and identification of pathogenic taxa typically rely on high-throughput metabarcoding of the small subunit ribosomal RNA gene (18S rRNA) (10). This approach enables the detection of diverse, low-abundance organisms. Despite its advantages, 18S-based gene profiling remains technically challenging due to amplification bias and primer-selection constraints. A persistent challenge in host-associated microbiome studies is the co-amplification of host 18S sequences. These host sequences can dominate sequencing libraries and obscure the detection of low-abundance microbial eukaryotes (17, 18). In diarrheic calves, enteritis leads to severe intestinal sloughing and epithelial shedding due to inflammation, which drastically increases host DNA shedding in feces (19, 20). The elevated levels of host DNA reduce the effective sequencing depth available for microbial profiling (21–23). Consequently, standard 18S metabarcoding often yields a substantial proportion of host-derived reads rather than the target microbial sequences (24), especially in samples with low eukaryotic biomass (25). This overwhelming host contamination increases overall costs and masks low-abundance microbes by reducing the recovery of informative parasite sequences (26). For apicomplexan parasites such as *Cryptosporidium*, excessive host amplification may also reduce the recovery of informative parasite sequences and compromise detection sensitivity.

To address this limitation, mammal-blocking primers have been developed to selectively bind to host 18S rRNA gene regions and suppress their amplification during polymerase chain reaction (PCR) (18). This strategy enriches non-host eukaryotic sequences and improves the recovery of target taxa. Mammal-blocking primers have been successfully utilized in marine and wildlife studies (18, 26–29). However, their performance in livestock-associated samples remains poorly characterized. In calves infected with *Cryptosporidium*, exceptionally high oocyst shedding rates (2, 30), combined with intestinal inflammation and altered gut physiology (3, 31), may further influence amplification efficiency and taxonomic recovery. These factors could distort our understanding of the infected gut ecosystem and hinder accurate characterization of eukaryotic community structure. To improve disease surveillance, it is vital to establish molecular frameworks capable of accurately profiling both apicomplexan pathogens and the surrounding microeukaryotic communities from field samples. Despite the increasing use of blocking primers in various studies (32–36), their utility for profiling the bovine gut eukaryome, particularly in diarrheic calves infected with *Cryptosporidium*, has not been systematically evaluated.

Interactions between parasitic organisms and resident microbial communities may influence host health and disease outcomes (16, 37), yet the eukaryotic microbiota of *Cryptosporidium*-infected calves remains poorly characterized. To address this gap, this study aimed to (i) evaluate four 18S rRNA primer sets (V8f/1510R, TAReuk454FWD1/TAReukREV3, 528F/706R, and 616F/1132R) with two mammal-blocking primers to identify a suitable primer-blocking strategy based on host-read suppression and eukaryotic taxonomic recovery, and (ii) apply the optimized workflow to characterize and compare the fecal eukaryotic communities of healthy calves and calves naturally infected with *C. parvum*.

## Materials and methods

### Experimental design and fecal sample collection

Fecal samples were obtained from calves (1 month old or less) from seven prefectures (eight regions) across Japan (Aichi, Ehime, Fukuoka, Hokkaido (Obihiro, Shihoro), Shizuoka, Yamagata, and Yamanashi). The collection period spanned from July 2024 to December 2025. The calves included both dairy breeds, primarily Holstein Friesians, and beef breeds, mainly the Japanese Black cattle. Sampling was performed by directly collecting feces from the calves’ rectums. Detailed metadata were recorded for each animal, including calf age, sex, breed, and geographical location, and are summarized in Supplementary Table 1. Within each region, both Healthy and Crypto calves were sampled from the same farm, thereby minimizing confounding by farm location within regions. Detailed information on feeding practices, housing, and farm management was not consistently available across all sampling sites and therefore could not be included as covariates in the analysis. Healthy control samples were selected based on clinical history and the absence of clinical signs, diarrhea, and *C. parvum* infection. During the pre-weaning period, calves exhibiting diarrhea and confirmed positive via GP60 PCR were classified as diseased (hereafter referred to as Crypto; *n* = 24), whereas calves without diarrhea that tested PCR-negative were classified as healthy (hereafter referred to as Healthy; *n* = 28) (7). Each sample was placed in an individually labeled sterile polystyrene tube, transported frozen in an isothermal container, and stored at −30 °C until DNA extraction.

### Use of ICT kits to screen *Cryptosporidium-*positive samples

Fecal samples were screened for *Cryptosporidium* antigen using the DipFit *Cryptosporidium* spp. ICT (Immunochromatographic Technique) kit (BIO K 387; Bio-X Diagnostics, Rochefort, Belgium) according to the manufacturer’s instructions. Briefly, a spoonful of feces was diluted and homogenized with the liquid provided in the kit. Precautions were taken to avoid foam formation. The ICT sticks were dipped in the homogenized mixture for 10 min. Positive samples showed successful formation of a band (Supplementary Figure 1).

### DNA extraction from fecal samples

Genomic DNA was isolated from fecal specimens using the QIAamp® Fast DNA Stool Mini Kit (Qiagen, Hilden, Germany). The manufacturer’s protocol was supplemented with a mechanical lysis step to ensure efficient disruption of the robust oocyst walls of *Cryptosporidium*. About 0.2 g of fecal material was combined with 1 mL of lysis buffer. After mixing with a wooden spatula and vortexing, the suspension was incubated at 70 °C for 10 min. Next, 0.30 μL of Proteinase K was added to the zirconia bead tube (EZ-Beads, Maxwell; Promega Corp., Madison, WI, USA). The pre-heated suspension of feces and lysis buffer was then transferred into this bead tube containing 0.30 μL of Proteinase K. The resulting mixture was thoroughly vortexed and subsequently centrifuged to collect the supernatant. The supernatant was transferred to a separate tube, 500 μL of 99.5% ethanol was added to the supernatant, briefly vortexed, and applied to the spin column for DNA binding. Subsequent wash steps were performed precisely as instructed by the manufacturer. To concentrate the final yield, the purified DNA was eluted in a reduced volume of 40 μL of AE elution buffer and stored at −20 °C for subsequent analysis.

### Amplification of the *Cryptosporidium gp*60 gene

Following immunochromatographic testing (ICT), extracted genomic DNA from fecal samples was used to perform a nested PCR targeting the 60-kDa glycoprotein (*gp*60) gene using primers designed for the specific amplification of *C. parvum*. Amplification was performed using the primer pairs AL3531 (5′-ATAGTCTCCGCTGTATTC-3′) and AL3535 (5′-GGAAGGAACGATGTATCT-3′) for the primary PCR, followed by AL3532 (5′-TCCGCTGTATTCTCAGCC-3′) and AL3534 (5′-GCAGAGGAACCAGCATC-3′) for the nested PCR, according to the protocol described by Alves et al. (38) and subsequently adopted in later studies ((39–41).

Both primary and nested PCR reactions were performed in a final volume of 25 μL using KOD FX NEO DNA polymerase (TOYOBO Co., Ltd., Osaka, Japan). Each reaction contained 1 μL of template DNA for the primary PCR or 1 μL of the primary PCR product for the nested PCR, 2.5 μL of 10 × KOD PCR buffer, 2.5 μL of dNTPs (2 mM), 1.5 μL of MgSO₄ (25 mM), 0.5 μL of each primer (25 μM), 16 μL of double-distilled water, and 0.5 μL of KOD amplification enzyme (1 U/μL). PCR amplification consisted of an initial denaturation at 94 °C for 3 min, followed by 35 cycles of denaturation at 94 °C for 45 s, annealing at 50 °C for 45 s, and extension at 72 °C for 1 min, with a final extension at 72 °C for 7 min. The nested PCR used the same cycling conditions with 1.0 μL of the primary PCR product as template. To ensure reproducibility, all samples were run in duplicate. A known *C. parvum* DNA sample was included as a positive control, and a no-template control containing nuclease-free water was included as a negative control in each PCR run. PCR products were analyzed by electrophoresis on a 1.5% agarose gel, and samples producing the expected nested PCR amplicon (~850 bp) were considered positive for *C. parvum*. Gels were stained with GelRed® (Biotium) and visualized on a UV transilluminator using the QIAxcel Advanced system (Qiagen, Valencia, California).

### Application of mammal-blocking primers to overcome the host DNA problem

To reduce the amplification of host-derived bovine 18S rRNA gene sequences and enhance the detection and resolution of low-abundance gut microbial eukaryotic taxa (including parasitic protozoa and commensal fungi), sequence-specific mammalian-blocking primers were incorporated into the 18S rRNA PCR amplification assays (18). The blocking primers were custom-designed to target highly conserved regions of the bovine (*Bos taurus*) 18S rRNA gene, operating via competitive elongation inhibition. All blocking oligonucleotides were modified at their 3′ termini with a three-carbon spacer group (/3SpC3/) (42). This prevents DNA polymerase from extending the host-bound strand, effectively halting amplification of the host DNA. At the same time, it allows target eukaryotic primers to anneal freely to microbial templates. Two distinct blocking primers were deployed based on the downstream hypervariable target region. A primer-specific blocking oligonucleotide (Ushi_18S_V8f_1510R_block) was used for the V8-V9 primer set (V8R/1510R), hereafter referred to as “V8.” This block targets sequences flanking the V8 region, minimizing host background during deep sequencing of this understudied domain. For assays targeting the V4 and V4-V5 regions, a universal mammalian blocking primer (Ushi_18S_block) was used for the TAReukF454/TAReukREV3, hereafter referred to as TAR; 528F/702R, hereafter referred to as 528; and 616F/1132R, hereafter referred to as 616 primer sets (Table 1). For each primer set, reactions were performed in the presence or absence of a mammal-blocking primer, designated as the Blocked and No block groups, respectively. To ensure a direct, paired evaluation across all assays, the same DNA extracts derived from four representative calf fecal samples (designated as individual biological replicates S1-S4; *n* = 4) were used as templates across all four primer sets (V8, TAR, 528, and 616) under both Blocked and No block conditions.

**Table 1:** Target primer sets, hypervariable regions, and mammal-blocking oligonucleotides used in this study. Four primer pairs targeting different hypervariable regions (V4, V4-V5, and V8-V9) of the eukaryotic 18S rRNA gene, with corresponding primer sequences, amplicon sizes, target regions, references, and the mammal-blocking primer sequences used for each set (modified with a 3′-C3 spacer to prevent elongation).

No.	Primer name	Primer sequences (F/R) 5′-3′	Target size (bp)	Target region	References	Mammal blocking primer
1	V8f	ATAACAGGTCTGTGATGCCCT	~512	V8-V9	Amaral-Zettler et al. (65); Bradley et al. (66)	Ushi_18S_V8f_1510R_block ACCCCATTCGTGATGGGGATCGGGGATTGCAATTATTCCC/3SpC3/
	1510R	CCTTCYGCAGGTTCACCTAC				
2	TAReuk454FWD1	CCAGCASCYGCGGTAATTCC	~400	V4	Stoeck et al. (24)	Ushi_18S_block AAAATTAGAGTGTTCAAAGCAGGCCCGAGCCGCC TGGATA/3SpC3/
	TAReukREV3	ACTTTCGTTCTTGATYRA				
3	528F	GCGGTAATTCCAGCTCCAA	~390	V4	Cheung et al. (59); Han et al. (62); Estrada et al. (61)	Ushi_18S_block AAAATTAGAGTGTTCAAAGCAGGCCCGAGCCGCC TGGATA/3SpC3/
	706R	AATCCRAGAATTTCACCTCT				
4	616*F	TTAAARVGYTCGTAGTYG	~516	V4-V5	Hugerth et al. (64)	Ushi_18S_block AAAATTAGAGTGTTCAAAGCAGGCCCGAGCCGCC TGGATA/3SpC3/
	1132R	CCGTCAATTHCTTYAART				

### Selection criteria for 18S rRNA primer-blocking sets

Primer-blocking combinations were evaluated using three complementary criteria: (i) the magnitude and consistency of host-read suppression across samples, (ii) the breadth and specificity of eukaryotic taxonomic recovery, and (iii) the extent of off-target amplification. Host suppression was assessed using the relative and absolute abundances of Vertebrata reads in blocked versus unblocked libraries, with reproducibility evaluated across samples rather than by the maximum reduction observed in any individual sample. Eukaryotic recovery was assessed based on the number and range of eukaryotic phyla and genera detected after excluding host-derived and non-target prokaryotic sequences. Primer-blocking combinations were therefore not ranked solely according to the magnitude of host-read reduction; instead, the combination providing the most favorable overall balance, considering host suppression, reproducibility, eukaryotic recovery, and specificity, was selected for subsequent profiling. A comparative summary of these criteria and the performance of each primer-blocking combination is provided in Supplementary Table 2.

### Library preparation and sequencing

The V8 and V3-V4, and V4-V5 hypervariable regions of the eukaryotic 18S rRNA gene were targeted for amplicon sequencing. A crucial element of this protocol was the inclusion of a mammal blocking primer (Table 1) to eliminate contamination from host DNA. We employed a two-step, tailed PCR approach for amplification. The primer sequences, reaction mixture composition, and thermal cycling parameters for the initial PCR step, both with and without the mammal blocking primer across target regions, are summarized in Supplementary Table 3. The PCR products from both the first and second PCR steps were purified using VAHTS DNA Clean Beads (Vazyme, China), followed by a second amplification round. Library quality and fragment size distribution were subsequently assessed using a Fragment Analyzer and the dsDNA 915 Reagent Kit (Agilent Technologies, CA, USA). The qualified products were pooled and sequenced on a 2 × 300 bp Illumina MiSeq system using a MiSeq Reagent Kit v3 (Illumina).

### Bioinformatic processing and taxonomic filtering

Sequence analysis was conducted in QIIME 2 (version 2022.2) (43, 44). Primer sequences were trimmed using the Cutadapt plugin (45) with a maximum error rate of 0.1 (−-p-error-rate 0.1) and --p-discard-untrimmed True, which discards reads lacking the specified primer sequence. The forward primer sequence was V8f (5′-ATAACAGGTCTGTGATGCCCT-3′), and the reverse primer sequence was 1510R (5′-CCTTCYGCAGGTTCACCTAC-3′).

Subsequent sequence processing, which included denoising, quality filtering, dereplication, chimera removal, and merging of paired-end reads, was performed using the DADA2 plugin (46). During DADA2 processing, the first 25 bases were removed from both forward and reverse reads (−-p-trim-left-f 25 and --p-trim-left-r 25), and reads were truncated at 240 bases for forward reads and 220 bases for reverse reads (−-p-trunc-len-f 240 and --p-trunc-len-r 220). Amplicon sequence variants (ASVs) were subsequently generated from the denoised sequences.

Taxonomic assignment of the resulting 18S rRNA gene ASVs was performed using the q2-feature-classifier plugin (47) within the QIIME 2 framework (48, 49). Specifically, a pre-trained Naive-Bayes machine-learning classifier (silva-138-99-nb-classifier.qza) (50), trained on full-length sequences from the SILVA SSU database release 138 (51), was utilized to assign taxonomy to the eukaryotic sequences. Taxonomic assignments were performed using the default minimum confidence threshold of 0.7. To provide a comprehensive overview of amplicon recovery, all taxonomically assigned sequences, including those classified as host-derived bovine (*Bos*) sequences, plant-derived/dietary (Magnoliophyta), mitochondria, and chloroplasts, were initially retained in the dataset. These complete profiles were utilized in the relative abundance visualizations (Figures 1A–H; Figures 2A,B) to illustrate the full spectrum of DNA recovered from fecal samples across the Healthy (*n* = 28) and Crypto (*n* = 24) groups. For downstream ecological analyses, including alpha diversity, beta diversity, and differential abundance testing, host-derived bovine, plant-derived, mitochondrial, and chloroplast sequences were subsequently excluded using the QIIME 2 taxa filter-table plugin. This filtering step focused the ecological analyses on non-host, non-plant eukaryotic sequences relevant to the gut eukaryotic community. Representative ASV sequences of selected taxa were compared against the NCBI GenBank database using BLASTn to support the taxonomic assignments obtained with the SILVA-based Naive Bayes classifier (52).

**Figure 1:**
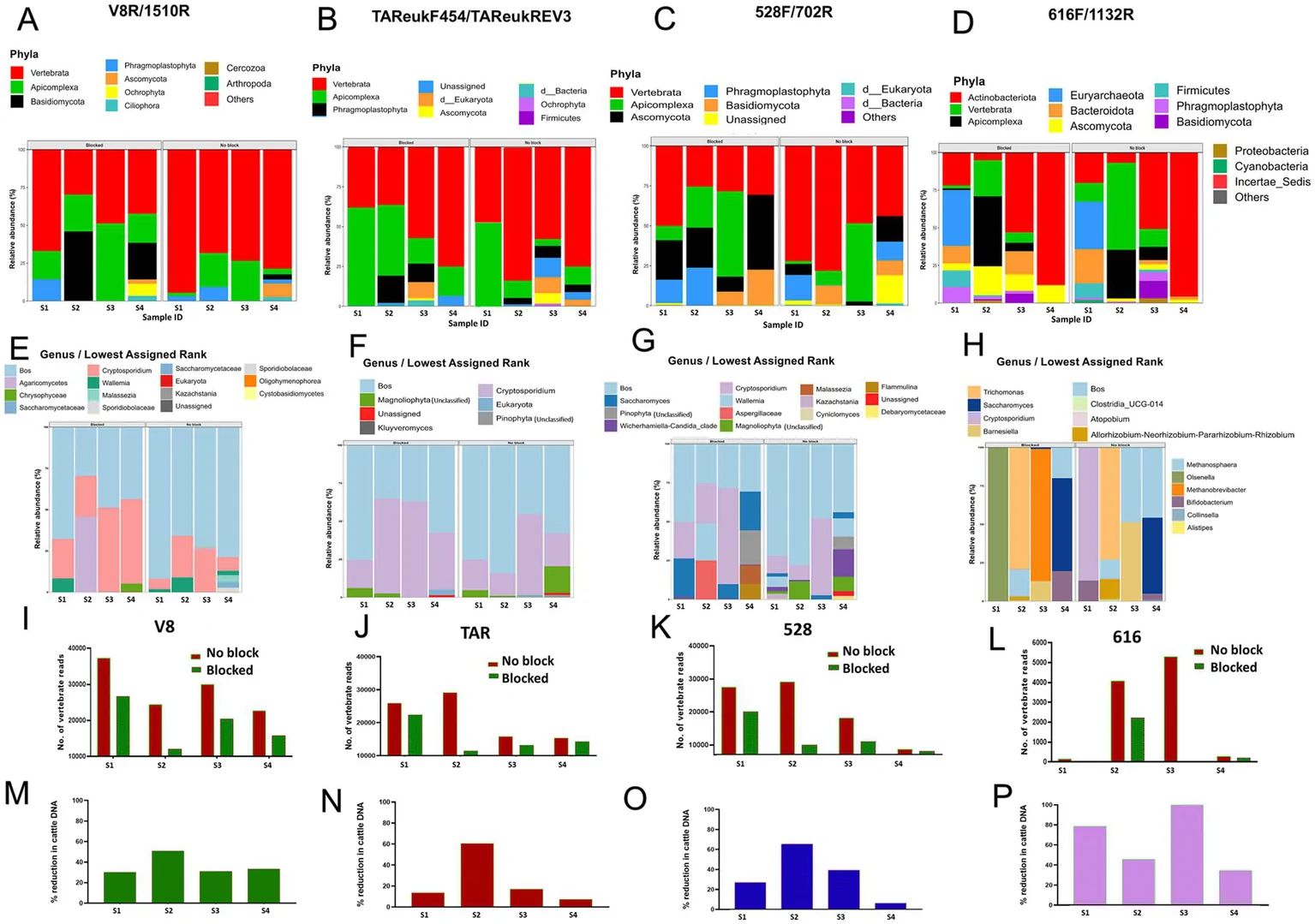
Comparison of taxonomic profiles and host DNA blocking efficiency across four 18S rRNA gene primer sets. Relative abundance of eukaryotic taxa at the **(A–D)** phylum level across four individual calf fecal samples (labeled S1–S4; *n* = 4, corresponding to biological replicates) using four distinct primer pairs under Blocked and No block conditions: **(A)** V8 (V8F/1510R), **(B)** TAR (TAReukF454/TAReukREV3), **(C)** 528 (518F/702R), and **(D)** 616 (616F/1132R). **(E–H)** Genus-level relative abundance (%) of identified taxa across samples for each respective primer set under Blocked and No block conditions. Panels **(I–L)** show the impact of host blocking primers on vertebrate read counts: Total number of vertebrate-assigned sequencing reads in the presence (Mammal block, green) versus absence (No block, red) of a mammalian blocking oligonucleotide across four experimental samples for each primer set. Panels **(M–P)** display the percentage reduction in host reads achieved by the blocking primer for each primer set. Taxa are color-coded by phylogenetic group, with low-abundance taxa pooled as “Others.” The V8f/1510R primer set demonstrated the most consistent host suppression and broadest recovery of eukaryotic diversity.

**Figure 2:**
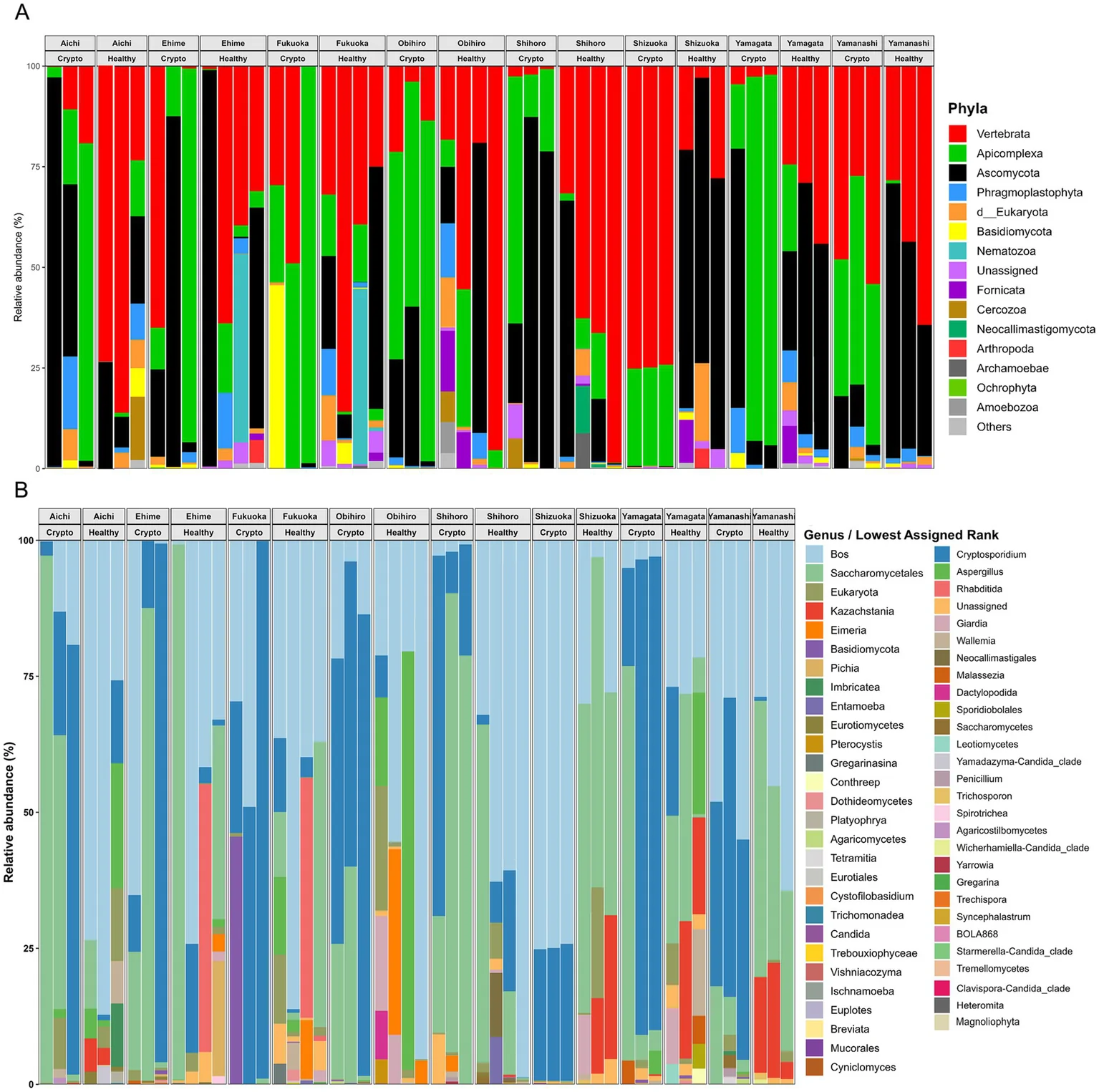
Eukaryotic community composition of the bovine gut microbiota in healthy and Cryptosporidium-infected calves. Relative abundance of eukaryotic taxa at the **(A)** phylum and **(B)** genus or lowest assigned rank in fecal samples from healthy (Healthy) and *Cryptosporidium*-positive diarrheic (Crypto) calves across eight Japanese prefectures (Aichi, Ehime, Fukuoka, Obihiro, Shihoro, Shizuoka, Yamagata, and Yamanashi). Vertical bars represent individual fecal samples collected from calves from distinct geographical locations. Taxa contributing less than 1% of total relative abundance are consolidated into the “Others” category. The phylum-level panel **(A)** highlights the dominance of Vertebrata (host-derived), Apicomplexa (parasitic protozoa), and Ascomycota/Basidiomycota (fungal communities). The genus-level panel **(B)** reveals pronounced enrichment of *Cryptosporidium* (dark blue/cyan) in the Crypto group, along with diverse fungal taxa (*Saccharomycetales*, *Aspergillus*, *Kazachstania*, *Pichia*), and residual host *Bos* sequences.

### Diversity and statistical analysis

Alpha- and beta-diversity analyses were performed using the QIIME 2 diversity plugin. Alpha-diversity metrics, including Shannon index, Simpson diversity, Faith’s Phylogenetic Diversity (PD), Observed Features (species richness), Pielou’s Evenness, and Chao1 richness, were calculated after rarefaction. For each primer set, samples were rarefied to the minimum sequencing depth appropriate for that dataset, as determined from rarefaction curve analysis. For downstream comparisons between Healthy and Crypto calves using the V8 primer set, samples were rarefied to 25,600 reads per sample.

Experimental comparisons were stratified by study design. For the comparative evaluation of the four 18S rRNA primer sets, where the same physical samples were processed under both Blocked and No block conditions (paired experimental design), alpha-diversity metrics were compared using the non-parametric Wilcoxon signed-rank test, and permutational multivariate analysis of variance (PERMANOVA) was performed using 9,999 restricted permutations within sample identity to account for repeated measures. For comparisons between distinct, independent groups of animals (Healthy vs. Crypto calves; two-group unpaired design), alpha-diversity metrics were evaluated using the non-parametric Wilcoxon rank-sum test.

Beta diversity was assessed using Bray-Curtis, Jaccard, and weighted UniFrac distance metrics, and principal coordinates analysis (PCoA) ordinations were generated and visualized using the phyloseq and ggplot2 packages in R. To evaluate the effects of clinical status (Healthy vs. Crypto) and geographical location on eukaryotic community composition, PERMANOVA was performed using the adonis2 function in the R package vegan (53) with 9,999 permutations (54, 55). A marginal (Type III) model was used to evaluate each factor while accounting for the other. Homogeneity of group dispersions was tested using permutational analysis of multivariate dispersions (PERMDISP) (56) via the betadisper function in vegan.

Phylum-level differential abundance between the independent Healthy and Crypto calf cohorts was assessed using the Wilcoxon rank-sum test. Only eukaryotic phyla detected at non-zero relative abundance in >10% of samples were retained for analysis, while host-derived taxa was explicitly excluded. *p*-values were adjusted for multiple comparisons using the Benjamini-Hochberg false discovery rate (FDR) method (57), with an FDR-adjusted *p* < 0.05 considered statistically significant. Cliff’s δ (delta) was calculated as a non-parametric effect-size measure to quantify the magnitude of differences between the two clinical groups.

Differentially abundant taxa across taxonomic ranks between Healthy and Crypto groups were further identified using Linear Discriminant Analysis Effect Size (LEfSe), with an absolute logarithmic LDA score > 2.0 and *p* < 0.05 considered significant (58). All statistical analyses and data visualizations were implemented in R version 4.4.2 (59) using the phyloseq, vegan (53), ggplot2, and rstatix packages, together with base R functions.

## Results

### Quality evaluation and sample statistics: healthy and crypto groups

We performed 18S rRNA gene amplicon sequencing of fecal samples collected from calves aged <1 month, divided into Healthy (*n* = 28) and Crypto groups (*n* = 24), to determine the changes in the calf fecal microbiota in response to the *C. parvum* infection. A total of 2,572,750 raw paired-end amplicon sequences were successfully generated from healthy and *C. parvum*-infected diarrheic fecal samples. Quality profile assessment revealed that most sequencing reads maintained high base-calling accuracy (Phred quality score >25) across the majority of nucleotide positions. We obtained a final curated dataset of 2,062,720 high-quality, non-chimeric sequences. With an average of 39,667 ± 4,057 sequences per sample, providing robust coverage for profiling the gut microeukaryotic community. The number of sequences in all samples ranged from 25,697 to 46,809 and were rarefied to a sequencing depth of 25,697 sequences per sample. Quality evaluation and sample statistics for the four primers are also shown in Table 2.

**Table 2:** Summary of high-throughput sequencing yield and quality filtering across target primer sets.

No.	Primer name	Raw reads	Non chimeric sequences	Average reads ± standard deviation	Reads range
1	V8f/1510R	396,808	312,988	39,123 ± 3,844	30,606-42,000
2	TAReuk454FWD1/TAReukREV3	395,140	261,130	32,641 ± 3,488	26,623-35,767
3	528F/706R	395,767	269,057	33,632 ± 8,256	17,620-41,506
4	616*F/1132R	380,311	112,915	14,114 ± 11,882	4,577-36,931

Comparison of total raw reads, non-chimeric sequences retained after quality control, average read counts per sample (mean ± standard deviation), and the range of sequence reads obtained using four distinct target primer pairs. The V8f/1510R primer set yielded the highest number of non-chimeric sequences and the most consistent read distribution, while the 616F/1132R primer set produced the lowest recovery and greatest variability.

### Selection criteria and comparative evaluation of 18S rRNA primer sets

To optimize eukaryotic profiling within the bovine gastrointestinal tract and mitigate the overwhelming amplification of host genomic DNA, four distinct 18S rRNA primer sets, V8 (V8R/1510R), TAR (TAReukF454/TAReukREV3), 528 (528F/702R), and 616 (616F/1132R), were evaluated under paired “Blocked” (blocking primer) and “No block” conditions (Figure 1).

The composition of the amplicon libraries revealed variations in recovered vertebrate reads (Figures 1I–L) and in blocking efficiency across the hypervariable regions (Figures 1M–P). The V8 primer exhibited consistent host suppression across the evaluated samples. Unblocked libraries were heavily dominated by host Vertebrata sequences (exceeding 75–90% relative abundance; 22,000–36,000 reads; Figure 1I). Addition of the mammalian-blocking primer reduced host-derived reads by approximately 30–50% across the evaluated samples (Figure 1M). Although greater reductions of approximately 60–65% were observed for TAR and 528 in individual samples, these reductions were not consistently reproduced across samples, with host-read reductions falling below 10% for TAR and 5% for 528 in sample 4 (Figures 1N,O). V8 showed the most consistent host-read reduction across the evaluated samples and recovered a broad range of target eukaryotic taxa. At the genus or lowest resolvable taxonomic level, V8 recovered the highest number of eukaryotic genera among the evaluated primer sets after excluding host-derived and prokaryotic sequences (Supplementary Table 2). Following host suppression, the libraries revealed substantial representation of the protozoan parasite *Cryptosporidium*, along with fungal genera including *Wallemia*, *Malassezia*, *Saccharomyces*, *Kazachstania*, and members of the *Agaricomycetes*. Additional low-abundance fungal and environmental taxa such as *Sporidiobolaceae*, *Cystobasidiomycetes*, and *Oligohymenophorea* were also detected, indicating that the V8 region captured a taxonomically diverse assemblage of gut-associated and environmental eukaryotes (Figure 1E). TAR and 528 primers displayed highly variable, sample-dependent blocking action. Both pairs achieved notable peak suppression in sample 2 (reducing cattle DNA by ~60% and ~65%, respectively (Figures 1N,O). However, this effect failed to replicate in sample no. 4, yielding negligible reductions (<10% for TAR; <5% for 528). Consequently, “Blocked” libraries still retained substantial host background contamination, and a large portion of the unmasked 528 reads belonged to Phragmoplastophyta (plant-derived) rather than protozoan lineages (Figure 1C). Genus-level profiles further highlighted differences between these primer sets. The TAR primer primarily recovered *Cryptosporidium* and plant-associated taxa, particularly Magnoliophyta and Pinophyta, together with the yeast *Kluyveromyces*. Compared with V8, TAR detected a considerably narrower spectrum of fungal and protistan genera (Figures 1E,F). The 528 primer set recovered *Cryptosporidium* and *Saccharomyces* but was additionally characterized by abundant plant-derived sequences and several fungal taxa, including *Wicherhamiella*-*Candida* clade members, *Aspergillaceae*, *Malassezia*, *Debaryomycetaceae*, and *Cyniclomyces*. Despite this broader fungal recovery, host-derived *Bos* sequences remained dominant in many samples, limiting the effective detection of parasitic protists (Figure 1G). Primer 616 demonstrated poor compatibility with the mammal-blocking design. While low baseline host amplification inflated percentage reductions in some samples (~80–100%) (Figure 1P), the corresponding unblocked host read counts did not exceed 5,500 (Figure 1L). In sample 2, it managed ~40% reduction (Figure 1P), frequently unmasking non-target bacterial phyla (*Actinobacteriota* and *Firmicutes*) and indicating a profound loss of eukaryotic specificity (Figure 1H). This loss of specificity was even more evident at the genus level. The 616F/1132R libraries contained high proportions of bacterial sequences, including *Methanobrevibacter*, *Methanosphaera*, *Olsenella*, *Bifidobacterium*, *Collinsella*, and other prokaryotic taxa. Although low levels of *Cryptosporidium*, *Trichomonas*, and *Saccharomyces* were detected, these target microeukaryotes represented only a minor component of the community (Figure 1L).

### Eukaryotic community composition across four primer sets

In addition to differences in host suppression efficiency, the four primer sets differed markedly in the breadth of eukaryotic taxa recovered. V8 recovered the broadest diversity of target eukaryotic phyla (Figure 1A), including *Apicomplexa*, *Basidiomycota*, *Ascomycota*, *Cercozoa*, *Ciliophora*, and *Ochrophyta*, and detected a diverse assemblage of genera such as *Cryptosporidium*, *Wallemia*, *Malassezia*, and *Saccharomyces*. TAR and 528 recovered fewer protistan and fungal taxa and were dominated by Vertebrata and plant-derived sequences in several samples (Figures 1B,C). Although the 616 primer detected the largest number of total phyla, these were predominantly bacterial and archaeal lineages, including *Actinobacteriota*, *Firmicutes*, *Proteobacteria*, *Bacteroidota*, and *Euryarchaeota*, indicating substantial off-target amplification and reduced eukaryotic specificity (Figure 1D). Collectively, genus-level analyses corroborated the phylum-level observations. V8 provided the most balanced recovery of parasitic protists and fungal taxa while maintaining effective suppression of host genus *Bos* between Blocked and No block groups (Figure 1E), TAR and 528 yielded intermediate performance with substantial plant and host contamination (Figures 1F,G), and 616 showed extensive amplification of bacterial and archaeal taxa, rendering it unsuitable for comprehensive eukaryotic community characterization (Figure 1H).

### Alpha and Beta diversity dynamics across four primer sets

To evaluate the effect of host DNA blocking on eukaryotic community metrics across different 18S rRNA primer pairs, alpha- and beta-diversity metrics were compared between host-blocked and non-blocked libraries across four candidate primer sets (V8f/1510R, TAReuk454FWD1/TAReukREV3, 528F/706R, and 616F/1132R). Overall, host DNA blocking did not result in statistically significant differences in alpha- or beta-diversity metrics for any primer set (*p* > 0.05 for all comparisons: Tables 3, 4). However, numerical changes in several alpha-diversity indices, particularly richness and evenness, were observed following host DNA blocking.

**Table 3:** Comparison of alpha diversity metrics between host DNA-blocked and non-blocked samples across four target primer sets.

Alpha diversity metrics	V8f/1510R			TAReuk454FWD1/TAReukREV3			528F/706R			616F/1132R		
	Blocked	No block	*p* value	Blocked	No block	*p* value	Blocked	No block	*p* value	Blocked	No block	*p* value
Simpson diversity	0.574	0.414	0.143	0.517	0.585	0.806	0.748	0.551	0.462	0.922	0.898	0.479
Chao1 diversity	17.8	13.2	0.834	27	10	0.101	10.2	20.5	0.306	45	54.66	0.288
Observed Features	17.8	13.3	0.834	27.5	10.2	0.101	11	20.5	0.306	18	15	0.422
Shannon Index	1.759	1.312	0.601	1.737	1.828	1	2.264	1.889	0.634	4.353	4.287	0.723
Pielou’s Evenness	0.554	0.397	0.050	0.501	0.357	0.147	0.667	0.450	0.220	0.790	0.768	0.479
Faith PD	3.805	8.294	0.065	13.88	4.89	0.220	9.990	4.01	0.141	4.031	9.04	0.172

Values present community richness, evenness, and phylogenetic diversity metrics (Simpson, Chao1, Observed Features, Shannon, Pielou’s Evenness, and Faith’s PD) for each primer pair under blocked and non-blocked conditions. P-values were calculated using the non-parametric Wilcoxon signed-rank test to account for the paired experimental design (same physical samples processed under both conditions). While no comparisons reached statistical significance (*p* > 0.05 for all metrics), the V8f/1510R primer set showed a borderline improvement in Pielou’s Evenness following blocking (*p* = 0.050).

**Table 4:** Beta diversity metrics for four 18S rRNA primer sets under Blocked and no-block conditions.

Beta diversity metrics	V8f/1510R			TAReuk454FWD1/TAReukREV3			528F/706R			616F/1132R		
	PERMANOVA (*R*^2^)	PERMANOVA (*p*)	PERMDISP (*p*)	PERMANOVA (*R*^2^)	PERMANOVA (*p*)	PERMDISP (*p*)	PERMANOVA (*R*^2^)	PERMANOVA (*p*)	PERMDISP (*p*)	PERMANOVA (*R*^2^)	PERMANOVA (*p*)	PERMDISP (*p*)
Bray-Curtis PCoA	0.091	0.416	0.244	0.127	0.353	0.075	0.170	0.127	0.063	0.0784	0.462	0.109
Jaccard distance	0.091	0.416	0.198	0.108	0.696	0.582	0.115	0.723	0.386	0.117	0.474	0.286
Weighted UniFrac PCoA	0.145	0.25	0.242	0.177	0.238	0.875	0.142	0.279	0.597	0.082	0.45	0.235

PERMANOVA (*R*^2^ and *p*-values) and PERMDISP (*p*-values) tests were performed across four target primer sets based on Bray–Curtis dissimilarity, Jaccard distance, and Weighted UniFrac distance metrics (*p* < 0.05 considered statistically significant). The V8f/1510R primer set showed the highest Bray-Curtis separation (*R*^2^ = 0.170). No significant differences were observed for any primer set (*p* > 0.05).

For the V8f/1510R primer set, host blocking maintained statistically comparable richness and diversity relative to unblocked controls, including Simpson diversity (0.574 vs. 0.414, *p* = 0.143), Chao1 richness (17.8 vs. 13.2, *p* = 0.834), Observed Features (17.8 vs. 13.3, *p* = 0.834), and Shannon Index (1.759 vs. 1.312, *p* = 0.601). Pielou’s evenness increased from 0.397 to 0.554 (*p* = 0.050), whereas Faith’s PD decreased from 8.294 to 3.805 (*p* = 0.065) (Table 3). Beta diversity analyses revealed that community structure for V8f/1510R remained comparable between blocked and unblocked conditions. Bray–Curtis dissimilarity showed the highest proportion of explained variance among the evaluated primer sets (PERMANOVA, *R*^2^ = 0.170, *p* = 0.116), with homogeneous group dispersion (PERMDISP, *p* = 0.244). Jaccard (*R*^2^ = 0.098, *p* = 0.206; PERMDISP, *p* = 0.198) and weighted UniFrac (*R*^2^ = 0.145, *p* = 0.250; PERMDISP, *p* = 0.242) analyses likewise showed no significant differences in community composition or dispersion between blocked and non-blocked libraries (Table 4).

For the TAReuk454FWD1/TAReukREV3 primer set, blocking resulted in numerically higher Chao1 richness (27.0 vs. 10.0, *p* = 0.101), Observed Features (27.5 vs. 10.2, *p* = 0.101), Pielou’s evenness (0.501 vs. 0.357, *p* = 0.147), and Faith PD (13.88 vs. 4.89, *p* = 0.220), whereas Simpson diversity (0.517 vs. 0.585, *p* = 0.806) and Shannon Index (1.737 vs. 1.828, *p* = 1.000) remained comparable. Beta-diversity analyses showed no significant differences among blocked and non-blocked libraries (Bray-Curtis *R*^2^ = 0.091, *p* = 0.244; Jaccard *R*^2^ = 0.177, *p* = 0.875; weighted UniFrac *R*^2^ = 0.108, *p* = 0.582). For the 528F/706R primer set, alpha diversity metrics were likewise comparable between blocked and unblocked samples, including Simpson diversity (0.748 vs. 0.551, *p* = 0.462), Chao1 richness (10.2 vs. 20.5, *p* = 0.306), Observed Features (11.0 vs. 20.5, *p* = 0.306), Shannon Index (2.264 vs. 1.889, *p* = 0.634), Pielou’s evenness (0.667 vs. 0.450, *p* = 0.220), and Faith PD (9.990 vs. 4.010, *p* = 0.141). Bray-Curtis analysis showed the greatest separation for this primer pair (*R*^2^ = 0.175, *p* = 0.163), whereas Jaccard (*R*^2^ = 0.115, *p* = 0.723) and weighted UniFrac (*R*^2^ = 0.142, *p* = 0.279) showed no significant differences. The 616F/1132R libraries showed relatively high alpha-diversity estimates, despite substantial representation of bacterial and archaeal sequences. Host DNA blocking did not significantly affect Simpson diversity (0.922 vs. 0.898, *p* = 0.479), Chao1 richness (45.00 vs. 54.66, *p* = 0.288), Observed Features (18.0 vs. 15.0, *p* = 0.422), Shannon Index (4.353 vs. 4.287, *p* = 0.723), Pielou’s evenness (0.790 vs. 0.768, *p* = 0.479), and Faith PD (4.031 vs. 9.040, *p* = 0.172). Beta-diversity analyses likewise showed no significant differences in community composition across Bray-Curtis, Jaccard, and weighted UniFrac distance metrics (PERMANOVA, all *p* > 0.400) (Tables 3, 4). Overall, host DNA blocking did not significantly affect alpha- or beta-diversity metrics for any of the evaluated primer sets. These analyses therefore provided no evidence of substantial community-level distortion following host blocking. Rather, differences among primer sets in host suppression, taxonomic recovery, and off-target amplification were the primary considerations in selecting the V8f/1510R combination for subsequent profiling.

### Composition of the eukaryotic fecal microbiota in healthy and *Cryptosporidium*-diarrheic cattle

To evaluate the eukaryotic community structure and assess the detection efficacy of the optimized V8 18S rRNA gene profiling platform, this study analyzed ASVs from 52 samples. Taxonomic assignments were examined at the phylum and genus (or lowest available taxonomic) levels across seven Japanese prefectures (Aichi, Ehime, Fukuoka, Hokkaido (Obihiro, Shihoro), Shizuoka, Yamagata, and Yamanashi). Samples within each region were categorized by clinical status into *Cryptosporidium*-infected (“Crypto”) and asymptomatic (“Healthy”) groups (Figure 2).

### Phylum-level composition of eukaryotic fecal microbiota in healthy and *Cryptosporidium*-diarrheic cattle

Amplicon sequencing recovered 13 eukaryotic phyla across all samples, with low-abundance phyla (each comprising <1% of the total community) pooled into the “Others” category. At the phylum level, the eukaryotic community was dominated by Vertebrata, Apicomplexa, and Ascomycota/Basidiomycota (Figure 2A). The relative distribution of these phyla varied between the Healthy and Crypto groups. Despite the application of the mammal-blocking primer, Vertebrata sequences remained abundant in several samples, indicating amplification and detection of bovine-derived DNA. Among non-host eukaryotes, Apicomplexa represented a major proportion of the community in several Crypto samples, particularly those from Fukuoka, Obihiro, and Yamagata (Figure 2A).

Differential-abundance analysis identified significant differences in the relative abundance of Apicomplexa and Fornicata between Healthy and Crypto calves after Benjamini-Hochberg correction. Apicomplexa was significantly more abundant in Crypto calves than in Healthy calves (median, 24.90% vs. 4.23%; FDR-adjusted *p* = 0.011). Fornicata also differed significantly between groups (FDR-adjusted *p* = 0.026), although its median relative abundance was 0% in both groups. Ascomycota showed a lower median relative abundance in Crypto calves than in Healthy calves (6.72% vs. 22.5%), but this difference did not reach statistical significance after FDR correction (FDR-adjusted *p* = 0.0526). The complete statistical results, including effect sizes, are presented in Supplementary Table 4. Additional eukaryotic phyla, including Cercozoa, Ciliophora, Amoebozoa, Ochrophyta, Nematozoa, and Neocallimastigomycota, were also detected (Figure 2A). Phragmoplastophyta was likewise detected in several animals, contributing to the overall taxonomic diversity observed across the fecal samples.

### Taxonomic composition at the genus or lowest assigned rank in the fecal eukaryotic community of healthy and *Cryptosporidium*-diarrheic cattle

At the genus (or lowest resolvable taxonomic) level, after removal of unassigned reads, the fecal eukaryotic community showed marked compositional heterogeneity between the Healthy and Crypto groups (Figure 2B). High relative abundances of *Cryptosporidium* were observed across multiple Crypto samples (dark blue/cyan bars). Notably, calves from Fukuoka, Obihiro, Shizuoka, and Yamagata frequently showed *Cryptosporidium* as the dominant non-host taxon, occasionally comprising more than 50% of total assigned relative abundance (Figure 2B). Importantly, the *Cryptosporidium* detected in these samples was supported by two complementary approaches: (i) species-specific nested PCR targeting the *gp*60 gene, a widely used molecular marker for *C. parvum* and (ii) BLASTn analysis of the *Cryptosporidium* ASV sequences identified by the SILVA-based Naive Bayes classifier against the NCBI GenBank database. The high relative abundance of *Cryptosporidium* sequences detected by the 18S metabarcoding in the Crypto group was therefore concordant with the species-specific GP60 PCR results and BLASTn identification. Together, these analyses supported the identification of the detected *Cryptosporidium* sequences as *C. parvum*. Interestingly, low levels of *Cryptosporidium* sequences were also detected in a small number of Healthy calves. (e.g., Ehime, Fukuoka, and Yamagata). Several protozoan genera, including *Eimeria* (orange bars), *Giardia* (light pink bars), and *Entamoeba* (purple bars), were also detected, although generally at lower relative abundances (Figure 2B).

### Identification of commensal fungi and co-occurring taxa

Beyond *Cryptosporidium*, the optimized 18S sequencing platform recovered a diverse collection of fungal, protozoan, and dietary eukaryotic taxa (Figure 2B). Fungal communities were frequently represented by taxa assigned to Saccharomycetales (light sage-green bars), along with genera such as *Aspergillus* (bright green bars), which were detected in both Healthy and Crypto calves across multiple sampling locations, including Aichi, Ehime, and Shihoro (Figure 2B).

Additional taxa, including *Kazachstania* (orange-red bars) and *Pichia* (light brown bars), were detected at higher relative abundances in Healthy calves than in Crypto calves (Figure 2B). Genera such as *Candida*, *Wallemia*, and *Trichosporon* were detected across multiple regions and in both clinical groups (Figure 2B). Plant-associated taxa belonging to Phragmoplastophyta (e.g., Imbricatea lineages) were detected in multiple individuals across the study groups. Other anaerobic fungal taxa, such as *Neocallimastigales*, were identified in some samples. A substantial fraction of sequences across both groups were classified as *Bos* (light blue/teal bars), indicating the presence of residual host-derived sequences despite application of the mammal-blocking primer (Figure 2B).

### Influence of covariates on eukaryotic community composition

To determine whether geographic location confounded the observed differences between the Healthy and Crypto groups, we performed PERMANOVA with clinical status and geography as explanatory variables. In the marginal model, clinical status explained 12.91% of the variation (*R*^2^ = 0.1291, *p* = 0.0001), whereas geography accounted for 15.50% of the variation but was not statistically significant (*R*^2^ = 0.1550, *p* = 0.1798). PERMDISP showed no significant difference in multivariate dispersion between the Healthy and Crypto groups (*F* = 2.0105, *p* = 0.1624). These results indicate that clinical status was significantly associated with eukaryotic community differences after accounting for geographic variation, whereas the marginal effect of geography was not statistically significant.

### Alpha and Beta diversity of the eukaryotic fecal microbiota in healthy and *Cryptosporidium*-infected cattle

To assess the impact of clinical status on the intestinal microeukaryotic community, alpha-diversity metrics were compared between *Cryptosporidium*-infected and healthy calves. Alpha-diversity analyses revealed significantly lower Chao1 richness and Observed Features in the *Cryptosporidium*-infected group compared with healthy controls (both *p* = 0.00001) (Figures 3B,C). Shannon diversity was also significantly lower in infected animals (*p* = 0.041; Figure 3D). In contrast, Simpson diversity (*p* = 0.23), Pielou’s evenness (*p* = 0.76), and Faith’s PD (*p* = 0.47) did not differ significantly between groups (Figures 3A,E,F).

**Figure 3:**
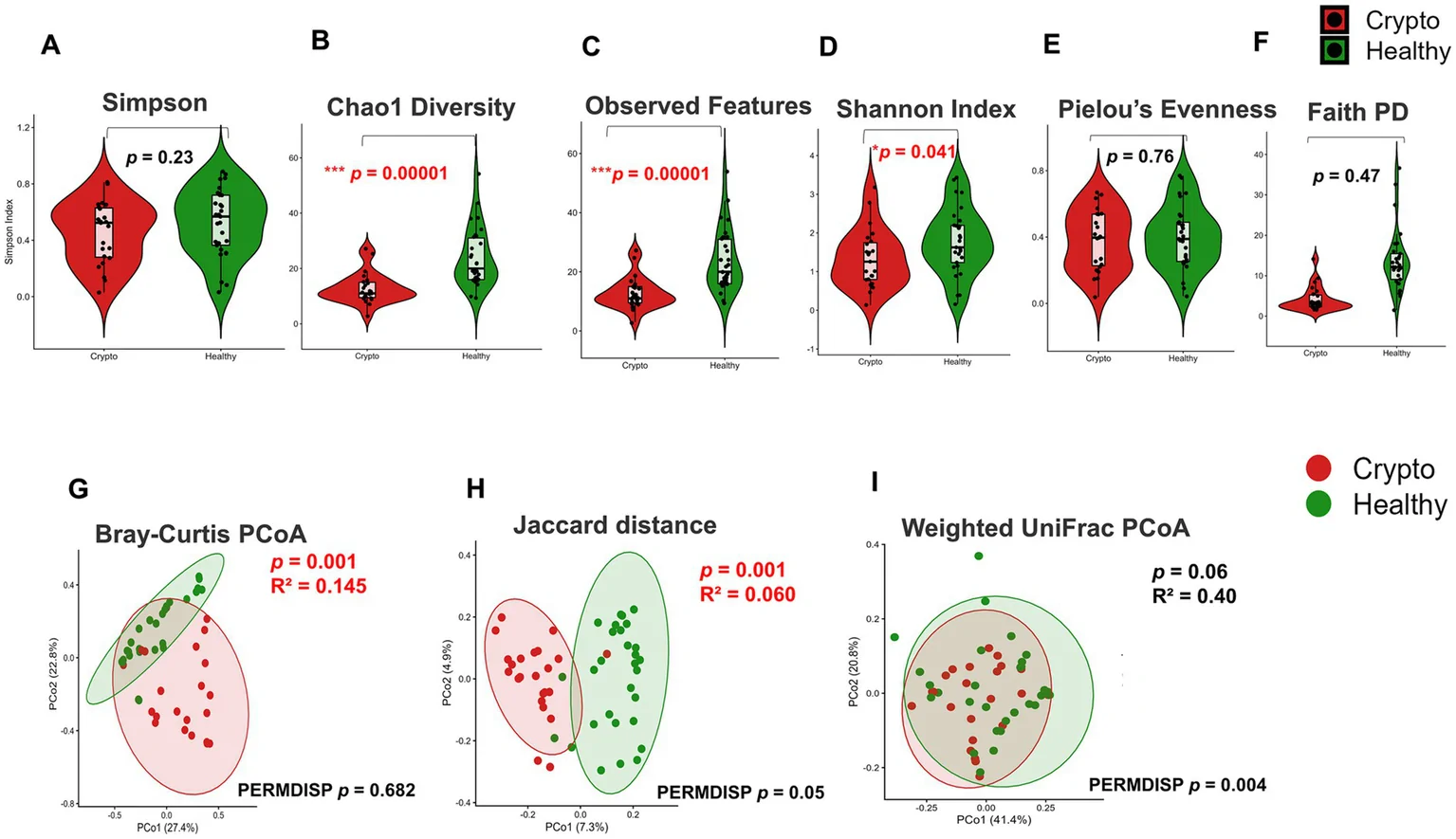
Alpha and beta diversity dynamics of gut eukaryotic communities in healthy and *Cryptosporidium*-infected calves. **(A–F)** Comparison of alpha-diversity metrics between healthy (Healthy, green; *n* = 28) and *Cryptosporidium*-infected (Crypto, red; *n* = 24) calves. Alpha diversity was assessed using the **(A)** Simpson Index (*p* = 0.23), **(B)** Chao1 Diversity (***p = 0.00001), **(C)** Observed Features (***p = 0.00001), **(D)** Shannon Index (*p = 0.041), **(E)** Pielou’s Evenness (*p* = 0.76), and **(F)** Faith’s PD; *p* = 0.47). Violin plots show the distribution of values within each group, with embedded boxplots representing the median and interquartile range. Statistical significance between two independent groups was evaluated using the non-parametric Wilcoxon rank-sum test. **(G–I)** Beta-diversity analyses of eukaryotic community composition. PCoA plots were generated using **(G)** Bray–Curtis dissimilarity, with PERMANOVA showing *R*^2^ = 0.145, *p* = 0.001, **(H)** Jaccard distance, with PERMANOVA showing *R*^2^ = 0.060, *p* = 0.001, and **(I)** Weighted UniFrac distance, with PERMANOVA showing *R*^2^ = 0.040, *p* = 0.06. Abbreviations: Crypto, *Cryptosporidium*-positive; Healthy, clinically healthy. Each point represents an individual calf fecal sample colored by clinical group. Shaded ellipses represent 95% confidence intervals. Community differences were calculated using PERMANOVA with 9,999 permutations; *R*^2^ and *p*-values are shown. Homogeneity of group dispersions was assessed using PERMDISP.

Beta-diversity analyses demonstrated significant differences in eukaryotic community composition according to disease status. PCoA based on Bray–Curtis dissimilarity revealed significant separation between the Crypto and Healthy groups (PERMANOVA, *R*^2^ = 0.145, *p* = 0.001). No significant difference in group dispersion was observed (PERMDISP, *p* = 0.682), indicating that the significant PERMANOVA result was not attributable to unequal dispersion between groups (Figure 3G). Jaccard distance analysis similarly corroborated these findings, showing significant differences in community composition between groups (*R*^2^ = 0.060, *p* = 0.001; Figure 3H), although the PERMDISP result was borderline (*p* = 0.05). In contrast, weighted UniFrac analysis did not show a statistically significant difference in abundance-weighted phylogenetic community composition between groups (*R*^2^ = 0.040, *p* = 0.06). Notably, PERMDISP indicated significantly different within-group dispersion for weighted UniFrac (*p* = 0.004; Figure 3I), further limiting interpretation of the non-significant PERMANOVA result.

### Identification of differentially enriched eukaryotic taxa using LEfSe analysis

We applied LEfSe analysis to identify differentially abundant eukaryotic taxa associated with *Cryptosporidium* infection status in cattle fecal samples. Taxa exhibiting significant differences between *Cryptosporidium*-positive (Crypto) and healthy groups were identified using a logarithmic LDA score threshold > 2 (log10). The LDA bar plot (Figure 4A) revealed distinct taxonomic signatures between the two groups. LEfSe identified *Cryptosporidium* (LDA score = 5.6) as the strongest discriminator in the Crypto group, while *Kazachstania* (LDA score = 4.35), *Giardia* (LDA score = 4.0), and *Aspergillus* (LDA score = 0.8) were enriched in healthy calves. PERMANOVA (Adonis) demonstrated that the overall eukaryotic community composition differed significantly between Crypto and Healthy cattle group (*R*^2^ = 0.221, *p* = 0.001). The abundance heatmap (Figure 4B) provided an overview of the relative abundance patterns of detected eukaryotic taxa across the two groups. Distinct clustering of abundance profiles highlighted the dominance of *Cryptosporidium* in diseased animals, whereas several fungal and protistan taxa, including *Giardia*, *Aspergillus*, and *Kazachstania*, showed higher relative abundances in healthy cattle.

**Figure 4:**
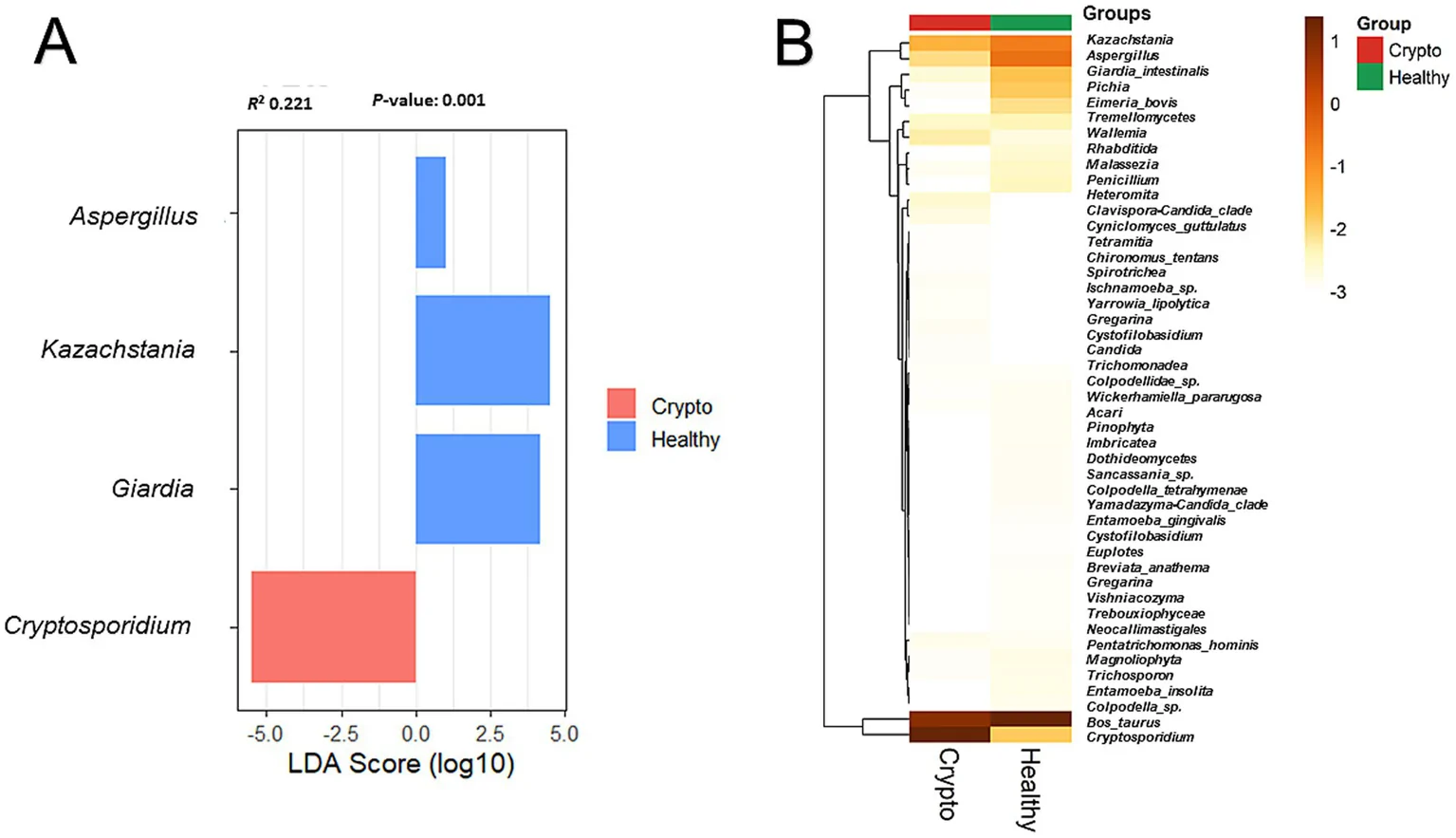
Linear discriminant analysis effect size (LEfSe) and differential abundance analysis of eukaryotic taxa between clinical cohorts. LEfSe analysis of the gut eukaryotic community structure at the genus level, used to identify eukaryotic taxa that differed significantly between Healthy and Crypto (LDA > 2, *p* < 0.05). **(A)** More abundant taxa in Healthy are shown in blue, and in Crypto are shown in red. Horizontal bars represent the effect size for each taxon. The length of the bar represents the log10-transformed LDA score, indicated by vertical lines. Overall community structure variance explained by clinical status is indicated by PERMANOVA (*R*^2^ = 0.221, *p* = 0.001). **(B)** Heatmap displaying relative abundances of detected eukaryotic taxa across the Crypto (red bar) and Healthy (green bar) groups.

## Discussion

Although several primer systems targeting different hypervariable regions have been developed and used in various studies, including V4 (24, 60–63), V4-V5 (64), and V8-V9 (65, 66). However, these studies have been predominantly limited to either soil protists, sediment eukaryotes, or marine environments rather than selective recovery of eukaryotes from host-rich samples (24, 62–64, 66). Furthermore, they were conducted without a mammal-blocking primer to avoid potential host contamination (61, 67, 68). Bradley et al. (66) reported limitations of commonly used V4 primers in mock-community analyses, whereas the V8-V9 region provided broad representation of the tested eukaryotic taxa. However, the performance of this region in host-rich bovine fecal samples, particularly when combined with mammalian host blocking, has not been well characterized. Therefore, our primer comparison provided an opportunity to assess whether the V8 region could provide a suitable balance between host suppression and recovery of fecal microeukaryotes.

The comparison of four 18S primer sets under blocked and unblocked conditions demonstrated that the effectiveness of mammalian host blocking depended on the primer region (Figure 1). Although the addition of the mammal-blocking primer reduced host-derived reads, the magnitude and consistency of suppression varied among primer sets, suggesting that successful host depletion depends on the compatibility between the blocking oligonucleotide, universal primers, and targeted 18S region (69). Among the evaluated combinations, V8 provided the most consistent host suppression together with broad recovery of target eukaryotic taxa, supporting its suitability for profiling the bovine fecal eukaryome (Figure 1). Nevertheless, residual *Bos* sequences remained abundant in several samples, indicating that host amplification could not be completely eliminated (Figure 2). This is consistent with other studies reporting variable blocking efficiencies with C3 spacer blockers across host species and sample types. For example, Liu et al. (29) reported that blocking primers with C3 spacers reduced host amplification in shrimp samples by 99%, but only 17% in oysters, whereas Huggins et al. (28) observed a 25% reduction in host mitochondrial DNA amplification in canine fecal samples. This variability likely reflects differences in host-template composition, primer design, and the degree of sequence complementarity between the blocking primer and the target DNA (26, 69). The variability in C3 spacer blocking efficiency may be attributed to incomplete inhibition of polymerase extension. DNA polymerase can displace or bypass the C3-modified blocking oligonucleotide, particularly when the blocking site is located relatively far from the PCR primer or when blocker-template binding is suboptimal (18, 29, 69). Thus, the principal advantage of the V8 combination in the present study was not complete host exclusion, but a reproducible reduction in host amplification while retaining broad eukaryotic taxonomic recovery.

Recently, peptide nucleic acid (PNA) clamp technology has emerged as an alternative strategy for host suppression and has generally demonstrated higher blocking efficiencies than conventional C3 spacer blockers. For example, Homma et al. (27) reported that PNA clamps suppressed 99.3–99.9% of host fish DNA, substantially outperforming conventional blocking primers, while Patchett et al. (26) similarly highlighted the superior host suppression achieved using PNA-based approaches. Nevertheless, C3 spacer blocking primers remain attractive because they are compatible with conventional DNA oligonucleotide synthesis, can be readily incorporated into standard PCR workflows, and are easily adapted for different host species (18, 29). Taken together, considering the balance between accessibility, flexibility, and sequencing performance, the V8 primer combined with the C3 spacer blocking primer represents a practical and effective approach for large-scale characterization of the bovine gut microeukaryome.

Ecological analyses provided additional insight into the performance of the V8 primer. Although TAR and 528 occasionally achieved greater host-read reductions in individual samples, these effects were not reproducible across samples. In contrast, V8 provided more consistent host suppression, together with broad recovery of eukaryotic taxa and limited off-target amplification. On this basis, the V8f/1510R combination was selected as the most suitable overall framework for subsequent profiling. Host DNA suppression was associated with preservation of richness and a trend toward improved evenness, suggesting that the V8-blocking combination enhances the recovery of microeukaryotic taxa without substantially altering overall community diversity or composition (Table 4). In contrast, the 528 primers showed more variable, sample-dependent host blocking, with substantial host contamination persisting in some samples. Although the 616 primer captured high overall richness (Table 3), its performance was compromised by extensive off-target amplification of bacterial taxa (Figures 1H,L,P), indicating that high richness estimates do not necessarily reflect eukaryotic diversity. Host blocking approaches require caution because mismatches between universal primers and blocking oligonucleotides may unintentionally suppress non-target taxa and introduce compositional bias (32, 69, 70). Despite these intrinsic risks, the V8f/1510R primer combined with host DNA blocking provided the most balanced performance among the evaluated primer sets, trending toward an effective compromise between host reduction and microeukaryotic recovery.

Using the optimized V8-mammal-blocking primer workflow, we characterized the fecal eukaryotic communities of healthy and *C. parvum*-infected calves, revealing marked differences in the relative abundance and composition of eukaryotic taxa across phylum and genus/lowest assigned taxonomic levels (Figure 5). The high relative abundance of *Cryptosporidium* in diarrheic calves is consistent with the heavy parasite burden associated with acute cryptosporidiosis (2, 30). Although cattle are susceptible to four major *Cryptosporidium* species: *C. parvum*, *C. bovis*, *C. ryanae*, and *C. andersoni*, only *C. parvum* is consistently associated with clinical disease in neonatal calves (30). Species-level confirmation using nested PCR targeting the *gp60* gene supported the identification of the apicomplexan signal in the diarrheic group as *C. parvum*. This confirmation reinforces the reliability of the 18S metabarcoding approach for detecting this clinically important enteric pathogen. Interestingly, low levels of *Cryptosporidium* sequences were also detected in a few healthy calves. This detection may indicate transient shedding or subclinical infections caused by less pathogenic species, such as *C. bovis* or *C*. *ryanae* (2, 30, 71)*.* Alternatively, it may reflect differences in pathogenicity among *Cryptosporidium* species and variation in host immune competence, both of which influence infection outcome and clinical presentation (72, 73). Notably, the detection of *Cryptosporidium* sequences in clinically healthy calves highlights the analytical sensitivity of the optimized V8-mammal-blocking workflow, enabling the identification of low-level or subclinical infections that may contribute to asymptomatic parasite circulation within cattle populations (30).

**Figure 5:**
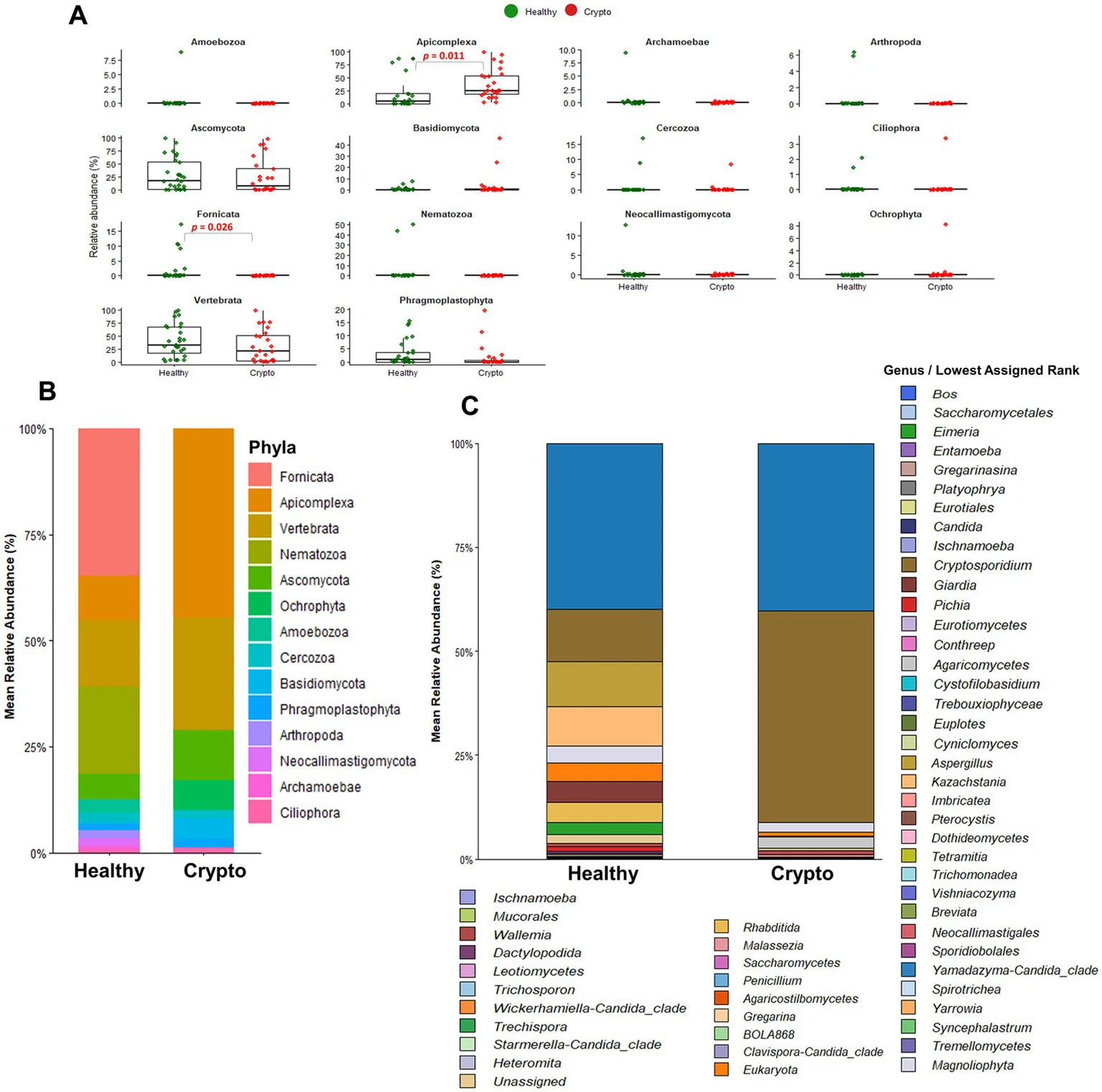
Mean relative abundance of fecal eukaryotic taxa in healthy and *Cryptosporidium*-infected calves. **(A)** Relative abundance (%) of detected eukaryotic phyla in individual calves, shown as box-and-whisker plots. Green points represent healthy calves and red points represent *Cryptosporidium*-infected calves. **(B)** Mean relative abundance (%) of detected fecal eukaryotic phyla or higher detected level in the healthy and *Cryptosporidium*-infected groups. **(C)** Mean relative abundance (%) of detected fecal eukaryotic taxa assigned to the genus level or, where genus-level assignment was unavailable, the lowest taxonomic rank assigned.

Extensive colonization of the intestinal epithelium by *C. parvum* can damage the intestinal mucosa, disrupt epithelial integrity, and alter the intestinal microenvironment (30, 74–76), thereby contributing to the restructuring of the resident gut microeukaryotic community. This pathogen-driven ecological shift is a classic feature of enteric pathogen-induced dysbiosis (77). Consistent with this, the observed reduction in Chao1 richness, Observed Features, and Shannon diversity in infected calves indicates a loss of taxonomic richness (Figures 3A–F). The divergence among alpha-diversity metrics is biologically meaningful. Chao1 and Observed Features, which primarily measure taxonomic richness, were reduced, whereas Simpson diversity and Pielou’s evenness showed no significant differences. This pattern suggests that while *C. parvum* infection led to a loss of eukaryotic taxa, the remaining community maintained a relatively balanced distribution without a single taxon becoming dominant (Figures 3A,E) (78–81). This differential response of richness and evenness indices underscores the importance of using multiple alpha-diversity metrics to capture distinct ecological facets of community change (82, 83). These findings are consistent with previous reports that gastrointestinal disease is frequently accompanied by loss of microbial diversity and altered community structure (7, 8, 21). Scully et al. (7) reported that alpha diversity increased with time in dairy heifer calves, but diarrheic calves had reduced microbial diversity on the day of sampling. Similarly, Slanzon et al. (8) found that differences in fecal microbiome composition were most evident in calves with gastrointestinal disease rather than between breeds. Importantly, infection-associated dysbiosis does not necessarily produce uniform changes across all aspects of diversity (84).

The observed beta-diversity patterns suggest that *C. parvum* infection was associated primarily with differences in taxonomic community composition rather than a uniform alteration of phylogenetic structure (Figures 3G–I). The strongest evidence was obtained with Bray–Curtis dissimilarity, for which the significant community separation was not accompanied by evidence of unequal dispersion. This supports the conclusion that the difference in community composition is attributable to a true biological shift rather than artifacts of unequal group variance (Figure 3G) (84). Jaccard analysis provided a similar indication of compositional differences, although its borderline PERMDISP result warrants more cautious interpretation because PERMANOVA can be influenced by differences in dispersion (Figure 3H; (85)). In contrast, weighted UniFrac did not detect a significant difference between groups, while its significant PERMDISP result indicated heterogeneous dispersion (Figure 3H). Thus, the weighted UniFrac result should be interpreted cautiously and does not provide evidence for a consistent disease-associated shift in abundance-weighted phylogenetic community structure. Taken together, these findings suggest that *C. parvum*-associated dysbiosis in the fecal eukaryotic community is more evident in taxonomic community composition than in the abundance-weighted phylogenetic structure captured by weighted UniFrac (86).

In addition to *Cryptosporidium*, the optimized 18S workflow recovered a broad diversity of fungal, protistan, and eukaryotic taxa, highlighting the complexity of the bovine gut microeukaryome (Figure 2B). The fungal community was dominated by the yeast-associated taxa, consistent with previous reports describing the ruminant mycobiome as being dominated by fungi belonging to the phyla Ascomycota and Basidiomycota (87, 88). This pattern reflects broader descriptions of the mammalian gut mycobiome as a low-diversity ecosystem enriched in *Saccharomyces*, *Candida*, and *Malassezia* (15, 89). Similarly, Ost and Round (15) noted that *Candida* and *Saccharomyces* commonly dominate the mammalian intestinal mycobiome, while environmental molds such as *Aspergillus* are also regularly detected. Although fungi constitute a relatively minor proportion of total intestinal biomass, commensal fungi contribute to epithelial homeostasis, immune modulation, and interkingdom microbial interactions (15, 90). Notably, *Kazachstania* and *Pichia* were abundant in healthy calves but declined markedly in the Crypto group. This reduction may reflect disruption of the normal fungal community following *C. parvum* colonization, either through competitive exclusion by the expanding parasite population or immune-mediated alterations of the intestinal niche during acute infection (15).

Visual examination of the relative taxonomic profiles across the eight sampling locations (Aichi, Ehime, Fukuoka, Obihiro, Shihoro, Shizuoka, Yamagata, and Yamanashi; Figure 2) revealed descriptive variation in fecal eukaryotic community composition. Differences were observed in the representation of major eukaryotic groups, including Apicomplexa, Ascomycota, Basidiomycota, and plant-associated Phragmoplastophyta, as well as lower-rank taxa such as *Cryptosporidium*, *Giardia*, and several fungal lineages. For example, variation in plant-derived reads, including Magnoliophyta, may reflect differences in dietary inputs, forage sources, or farm-specific management practices, although these factors were not systematically assessed in the present study (91, 92). Such variation is consistent with evidence that bovine fecal microbial communities are influenced by diet, feeding practices, and farm-level management (91, 93). Previous studies have also reported that both feeding management and geographical location can contribute to variation in bovine gut microbial communities (94, 95). However, these patterns should be interpreted cautiously because the present study was not designed to evaluate regional differences independently. In our multivariable PERMANOVA, geographical location accounted for 15.50% of the variation in community composition but was not significantly associated with community structure after accounting for clinical status (*R*^2^ = 0.1550, *p* = 0.1798). In contrast, clinical status remained significantly associated with community composition (*R*^2^ = 0.1291, *p* = 0.0001). This suggests that clinical status may have contributed more strongly to community variation than geographical location in the present dataset, consistent with previous studies showing that enteric pathogen infection can be associated with substantial gut dysbiosis (8, 96, 97). Thus, the regional patterns observed in Figure 2 should be regarded as descriptive heterogeneity rather than evidence of distinct geographic community structures and may reflect the contribution of clinical status, host characteristics, diet, environmental exposure, and unmeasured farm-level factors (98).

LEfSe analysis identified infection-associated taxonomic signatures, with *Cryptosporidium* representing the strongest discriminatory taxon in the Crypto group (Figures 4A,B). The enrichment of *Cryptosporidium*, together with the significant differences in overall community composition observed in the PERMANOVA, indicates that *C. parvum*-infected calves were characterized by distinct fecal eukaryotic community profiles. Interestingly, *Giardia* and *Aspergillus* were enriched in healthy calves. The enrichment of *Aspergillus* in healthy calves may reflect greater environmental exposure to this ubiquitous saprophytic fungus or differences in the gut mycobiota composition (89). Although *Giardia* is classically recognized as an enteric pathogen, its higher abundance in healthy calves is more likely to reflect differences in parasite co-colonization dynamics, host exposure history, or ecological interactions within the intestinal niche, rather than a direct protective effect against cryptosporidiosis (74, 99). This interpretation is supported by evidence that intestinal protozoa engage in complex ecological interactions with the fecal microbiota, and that infection outcomes can be strongly influenced by the surrounding microbial community (99, 100). Similar interactions have also been described among other apicomplexan parasites, in which infection with one species can influence the invasion or replication of another (101). The enrichment of multiple non-*Cryptosporidium* eukaryotes (*Giardia*, *Aspergillus*) further suggests that the observed community differences cannot be attributed exclusively to *C. parvum* and may instead reflect the combined influence of host, environmental, and microbial factors. Overall, our findings indicate that *C. parvum* infection is associated with broader restructuring of the bovine gut microeukaryome.

Several limitations warrant consideration and should be addressed in future studies. A methodological limitation of this study is that it was based exclusively on fecal samples. Although fecal samples provide a practical and non-invasive representation of the distal intestinal microbial community, they may not fully reflect eukaryotic communities in other gastrointestinal compartments, particularly the rumen, which has distinct physicochemical conditions and microbial ecology. Therefore, our findings are most appropriately interpreted as representative of the fecal eukaryotic community rather than the entire bovine gut eukaryome. Sampling across multiple gastrointestinal compartments would be required to determine whether the observed patterns extend beyond the fecal compartment. We acknowledge that detailed information on farm-level management practices, including feeding regimens, housing, and biosecurity, was not systematically collected. Although geographic variation was accounted for in the multivariable analysis, unmeasured factors, including breed distribution, feeding practices, farm management, environmental conditions, and potential co-infections, may have contributed to the observed microbial differences. Thus, the dysbiosis observed in this study should be interpreted as an association with *C. parvum* infection rather than evidence of a direct causal effect, as other host and environmental factors may also influence the fecal eukaryotic community (7, 8). Future longitudinal or experimental studies incorporating standardized management conditions and comprehensive host and environmental metadata will be necessary to disentangle the effects of *Cryptosporidium* infection from other sources of variation in the fecal eukaryotic community. Despite substantially reducing host-derived sequences, the C3 spacer blocking primer did not completely block host amplification, and residual bovine reads may have masked the detection of low-abundance eukaryotic taxa, particularly those sharing high sequence similarity with the host 18S rRNA gene. The C3 blocking strategy provided a practical balance between host suppression and taxonomic recovery; however, future research should evaluate peptide nucleic acid (PNA) clamp technologies, which have demonstrated superior host-blocking efficiency in other animal systems (26, 27). In addition, low-abundance *Giardia* and *Cryptosporidium* detections in healthy calves should be validated using species-specific PCR assays and confirmatory sequencing to distinguish true subclinical infections from potential methodological artifacts. Species-level molecular confirmation was performed only for *C. parvum* using a *C. parvum*-specific nested PCR assay targeting the *gp*60 gene in diarrheic calves. However, the resulting amplicons were not sequenced, limiting the ability to assign specific subtype families (e.g., IIa, IId). Future sequencing of this target would enable characterization of *C. parvum* subtype families, improve understanding of transmission dynamics, and facilitate assessment of the zoonotic potential of circulating strains, as several subtypes are associated with both human and bovine infections (102, 103). The remaining protozoan taxa detected by metabarcoding were not independently confirmed, limiting assessment of their epidemiological significance and pathogenic potential. It should also be noted that the DNA-based molecular methods used in this study, including 18S rRNA metabarcoding and GP60 PCR, cannot distinguish DNA derived from viable organisms from DNA originating from non-viable organisms or residual DNA. Therefore, detection of *C. parvum* and other eukaryotic taxa by 18S metabarcoding or PCR should not be interpreted as direct evidence of organismal viability or infectivity. This limitation is particularly relevant for *Cryptosporidium*, as DNA-based detection does not reliably establish oocyst viability (104, 105). Future studies incorporating viability-sensitive approaches, such as PMA-based PCR or RNA-based assays, could help distinguish viable from non-viable organisms and provide a more functionally relevant assessment of the fecal eukaryotic community. Consequently, the detected eukaryotic community may include DNA from organisms that are no longer viable, which should be considered when interpreting community composition and its association with infection status (106, 107). The fungal component of the bovine gut microeukaryome also warrants further investigation. Although 18S rRNA metabarcoding provided broad taxonomic coverage, ITS2 sequencing offers higher taxonomic resolution for fungal communities and would enable more detailed characterization of the bovine gut mycobiome (89). Finally, integrating bacterial 16S rRNA gene sequencing with 18S-based eukaryotic profiling would provide a more comprehensive understanding of cross-kingdom interactions within the bovine gut microbiome and help elucidate how *Cryptosporidium* infection influences bacterial, fungal, and protistan communities simultaneously (108).

## Conclusion

Overall, our findings reinforce the view that the bovine gut harbors a taxonomically diverse microeukaryotic community extending far beyond pathogenic protozoa. The optimized V8-mammal-blocking workflow enabled the recovery of a broad range of fungal, protistan, and eukaryotic taxa, thereby demonstrating its value for comprehensive profiling of the bovine gut eukaryome. Collectively, this approach will facilitate future investigations of host–parasite-microbiota interactions and support the development of improved surveillance strategies for livestock health.

## Data Availability

The datasets presented in this study can be found in online repositories. The names of the repository/repositories and accession number(s) can be found at: https://www.ddbj.nig.ac.jp/, The amplicon sequencing data generated in this study have been deposited in the DNA Data Bank of Japan (DDBJ) Sequence Read Archive (DRA) in FASTQ format under BioProject accession number PRJDB42773. Run and BioSample metadata are available under accession numbers DRR1076400-DRR1076455 and SAMD01945149–SAMD01945204, respectively. The original contributions presented in the study are included in the article. Further inquiries can be directed to the corresponding author.
