# Supplementary material for: In sickness and in health: profiling the bovine fecal eukaryome using optimized 18S metabarcoding with a mammal-blocking primer
Source: Front Vet Sci. 2026 Oct 8;13:1948426. doi: 10.3389/fvets.2026.1948426 (PMC13686857; doi:10.3389/fvets.2026.1948426)
Supplement: Supplementary file 1 [file Data_Sheet_1.docx]

Supplementary Material

**In sickness and in health: profiling the bovine fecal eukaryome using optimized 18S metabarcoding with a mammal-blocking primer**

**Iqra Zafar^1^, Riku Sakurai^1^, Yasuhiro Fukuda^1^, Megumi Itoh^2^, Atsushi Fujikura^3^, Akira Goto^4^, Kazuhiro Bandai^5^, Kentaro Kato^1*^**

^1^Laboratory of Sustainable Animal Environment, Graduate School of Agricultural Science, Tohoku University, Osaki, Miyagi 989-6711, Japan

^2^Department of Veterinary Medicine, Obihiro University of Agriculture and Veterinary Medicine, Obihiro, Hokkaido 080-8555, Japan

^3^Vets Support WA, Itoshima, Fukuoka, Japan

^4^Department of Veterinary Medicine, Faculty of Veterinary Medicine, Okayama University of Science, Ikoino-oka 1-3, Imabari, Ehime 794-0085, Japan

^5^Graduate School of Veterinary Science, Osaka Metropolitan University, Izumisano, Osaka 598-8531, Japan

*** Correspondence:**
Prof. Kentaro Kato D.V.M. Ph.D. Laboratory of Sustainable Animal Environment, Graduate School of Agricultural Science, Tohoku University, 232-3 Yomogida, Naruko-onsen, Osaki, Miyagi 989-6711, Japan.

Phone: +81-229-84-7391, Fax: +81-229-84-7391, E-mail: kentaro.kato.c7@tohoku.ac.jp
[kentaro.kato.c7@tohoku.ac.jp](mailto:kentaro.kato.c7@tohoku.ac.jp)

## Supplementary Figure 1. Detection of *Cryptosporidium* antigen using the DIPFIT® Crypto rapid diagnostic test.

(A) Representative negative result showing a single control line (indicated by an arrow). (B) Representative positive result showing both control and test lines (indicated by arrows).

**Supplementary Table 2. Comparative evaluation of 18S rRNA primer-blocking combinations.**

| **Criterion** | | | | | | **V8f/1510R** | **TAR** | **528F/706R** | **616F/1132R** |
| --- | --- | --- | --- | --- | --- | --- | --- | --- | --- |
| **Host-read reduction** | | | | | | **~30–50%** | **~60% in sample 2; <10% in sample 4** | **~65% in sample 2; <5% in sample 4** | **~40% in sample 2; apparent ~80–100% in some samples due to low baseline host reads** |
| **Consistency of host suppression** | | | | | | **Consistent** | **Variable** | **Variable** | **Variable / low baseline host amplification** |
| **Eukaryotic phyla recovered** | | | | | | **8** | **6** | **5** | **5** |
| **Eukaryotic genus-level recovery** | | | | | | **Broadest** | **Moderate** | **Moderate** | **Limited** |
| **Eukaryotic specificity** | | | | | | **High** | **Moderate** | **Moderate** | **Low** |
| **Off-target amplification** | | | | | | **Plant/host sequences** | **Plant/host sequences** | **Plant/host sequences** | **Extensive bacterial/archaeal sequences** |
| **Overall assessment** | | | | | | **Selected** | **Not selected** | **Not selected** | **Not selected** |
|  |  |  |  |  |  |  |  |  |  |

## Supplementary Table 3. Primer sequences, reaction mix compositions, and PCR thermal cycling conditions for first-round amplicon PCR under conditions with and without mammal blocking primers.

Reaction mixtures, primer details, and thermal cycling conditions, composition of first-round PCR reaction mixtures for eukaryotic 18S rRNA amplification. Four primer configurations were tested: (A) V8-V9 region with standard primers (V8F/1510R) without and with a blocking primer (Ush1_18S_V8F_1510R_block); and (B) V4-V5 region with standard primers (TAReuk454FWD1/TAReukREV3) without and with a blocking primer (Ush1_18S_block). The blocking primer in all "With Blocking Primer" conditions is a 3'-spacer-modified oligonucleotide designed to anneal to host (mammalian) 18S rDNA and prevent polymerase extension, thereby suppressing host-derived amplification. All reactions were performed in a total volume of 10.0 µL using KOD FX Neo polymerase. Abbreviations: dNTPs, deoxynucleotide triphosphates; /3SpC3/, 3-carbon spacer (blocking modification).

**Supplementary Table 4. Differential abundance of eukaryotic phyla between Healthy and *Cryptosporidium*-positive (Crypto) calves.** Relative abundances were compared between groups using the Wilcoxon rank-sum test. Only eukaryotic phyla detected at non-zero relative abundance in >10% of samples were included. *P*-values were adjusted for multiple comparisons using the Benjamini-Hochberg false discovery rate (FDR) method. Cliff's δ was calculated as a non-parametric effect-size measure. Adjusted *p* < 0.05 was considered statistically significant. Median relative abundances are expressed as percentages.

| **Phylum** | **Healthy median** | **Crypto median** | **Raw *p*** | **BH-adjusted *p*** | **Cliff's δ** |
| --- | --- | --- | --- | --- | --- |
| **Apicomplexa** | 4.23% | 24.90% | 0.00153 | **0.0110** | 0.441 |
| **Fornicata** | 0% | 0% | 0.00978 | **0.0261** | 0.360 |
| **Ascomycota** | 22.5% | 6.72% | 0.00927 | 0.0526 | 0.269 |
| Phragmoplastophyta | 1.01% | 0.025% | 0.0851 | 0.170 | 0.240 |
| Cercozoa | 0% | 0% | 0.118 | 0.189 | 0.219 |
| Basidiomycota | 0% | 0.065% | 0.193 | 0.220 | 0.182 |
| Vertebrata | 31.8% | 20.3% | 0.177 | 0.220 | 0.188 |
